# Antibiotic sales in rural and urban pharmacies in northern Vietnam: an observational study

**DOI:** 10.1186/2050-6511-15-6

**Published:** 2014-02-20

**Authors:** Do Thi Thuy Nga, Nguyen Thi Kim Chuc, Nguyen Phuong Hoa, Nguyen Quynh Hoa, Nguyen Thi Thuy Nguyen, Hoang Thi Loan, Tran Khanh Toan, Ho Dang Phuc, Peter Horby, Nguyen Van Yen, Nguyen Van Kinh, Heiman FL Wertheim

**Affiliations:** 1 Wellcome Trust Major Overseas Programme, Oxford University Clinical Research Unit, Hanoi, Vietnam; 2 Hanoi Medical University, Hanoi, Vietnam; 3 Vietnam National Cancer Hospital, Hanoi, Vietnam; 4 Department of Probability and Statistics, Institute of Mathematics, VAST, Hanoi, Vietnam; 5 Nuffield Department of Clinical Medicine, Centre for Tropical Diseases, Oxford, UK; 6 Hanoi Department of Health, Hanoi, Vietnam; 7 National Hospital for Tropical Diseases, Hanoi, Vietnam

**Keywords:** Antibiotic, Dispensing, Prescription, Community, Practice, Vietnam, Pharmacy

## Abstract

**Background:**

The irrational overuse of antibiotics should be minimized as it drives the development of antibiotic resistance, but changing these practices is challenging. A better understanding is needed of practices and economic incentives for antibiotic dispensing in order to design effective interventions to reduce inappropriate antibiotic use. Here we report on both quantitative and qualitative aspects of antibiotic sales in private pharmacies in northern Vietnam.

**Method:**

A cross-sectional study was conducted in which all drug sales were observed and recorded for three consecutive days at thirty private pharmacies, 15 urban and 15 rural, in the Hanoi region in 2010. The proportion of antibiotics to total drug sales was assessed and the revenue was calculated for rural and urban settings. Pharmacists and drug sellers were interviewed by a semi-structured questionnaire and in-depth interviews to understand the incentive structure of antibiotic dispensing.

**Results:**

In total 2953 drug sale transactions (2083 urban and 870 rural) were observed. Antibiotics contributed 24% and 18% to the total revenue of pharmacies in urban and rural, respectively. Most antibiotics were sold without a prescription: 88% in urban and 91% in rural pharmacies. The most frequent reported reason for buying antibiotics was cough in the urban setting (32%) and fever in the rural area (22%). Consumers commonly requested antibiotics without having a prescription: 50% in urban and 28% in rural area. The qualitative data revealed that drug sellers and customer’s knowledge of antibiotics and antibiotic resistance were low, particularly in rural area.

**Conclusion:**

Over the counter sales of antibiotic without a prescription remains a major problem in Vietnam. Suggested areas of improvement are enforcement of regulations and pricing policies and educational programs to increase the knowledge of drug sellers as well as to increase community awareness to reduce demand-side pressure for drug sellers to dispense antibiotics inappropriately.

## Background

Both appropriate and inappropriate use of antibiotics is a key driver of antibiotic resistance development. However, overuse or misuse of antibiotics (e.g. low dose, too short duration, or treatment of self-limiting infections) provides an avoidable additional pressure leading to more antibiotic resistance [1-3]. In many countries inappropriate use of antibiotics is common practice in the community setting, where antibiotics are readily dispensed for self-limiting upper respiratory tract infections without a prescription [4-8]. To slow down the development of antibiotic resistance, an important control strategy is to reduce the inappropriate use of antibiotics in both community and hospital settings. The incentives behind inappropriate antibiotic dispensing need to be fully understood, so intervention strategies can be developed based on that knowledge.

In Vietnam, health seeking behavior has changed since market reforms that were initiated since 1980s. Despite a public health care system, patients often bypass the health care system, and obtain medicines via self-medication or private pharmacies [9,10]. According to one study in 2002, the average household expenditure per episode of illness is 1.1 USD for self-treatment, 1.9 USD for private providers, and 5.2 USD for public providers. The relative higher costs of the health care system explain the preference for self-medication, which results in many cases of inappropriate drug use [10].

In Vietnam, legislation states that antibiotics can only be purchased with a medical prescription [11]. However, previous studies have shown that most antibiotics are sold without prescription. According to a community-based study undertaken in 1999, 78 percent of antibiotics were purchased in private pharmacies without a prescription. 67 percent of the participants consulted the pharmacist while 11 percent decided themselves about antibiotic use [12]. Only 27 percent of the pharmacy staff had correct knowledge about antibiotic use and resistance [8]. Reportedly, prevalence of self-medication with antibiotics through private pharmacies in rural Vietnam is 80%, and is even higher in children with 88% of the children receiving self-medication before hospital visit [13]. These results raised concerns about drugs being sold without prescriptions and the common practice of self-medication. Judicious use of antibiotics can decrease unnecessary adverse effects of antibiotics as well as out-of-pocket costs to the patient. But more importantly, decreased antibiotic usage will help delay the rise of drug resistant bacteria, which is now a growing world-wide public health problem [3].

The present study aims to understand the economic and behavioral incentives that support inappropriate dispensing of antibiotics at Vietnamese private pharmacies. This is crucial for designing effective interventions to reduce the inappropriate antibiotic use in the community.

## Methods

### Study sites and selection of pharmacies

The study was conducted at two well-established demographic surveillance sites (DSS) in the Hanoi region in 2010. The two study sites are: Bavi (site name: FilaBavi [14]) and Dong Da (site name: Dodalab [15]). Bavi is a rural community situated 60 km west of Hanoi. The basic health care system includes a district hospital with 150 beds, 3 regional polyclinics, 32 commune health stations, and 90 licensed private health facilities including private clinics, pharmacies, drug stores and drug outlets [16]. Dong Da is the biggest urban district of Hanoi with the public health care system including a district hospital with 300 beds, 3 regional polyclinics, 1 antenatal clinic, 21 commune health stations and 278 private pharmacies located in this district [15].

Private pharmacies in each site were randomly selected from a government pharmacy registry, using the Excel random number function for the rural and urban settings. The randomly selected pharmacies were approached sequentially based on ascending random number allocation to get permission to participate until 15 shops in each site were reached. All pharmacies that agreed to participate in the study allowed observers in their pharmacy during three days to observe and record drug sales and prices.

### Sample size

One of the major areas of focus in this cross-sectional study was the selling of antibiotics without a prescription and the revenue of antibiotic sales as compared to all sales. Based on previous work we expected 80% of customers to buy antibiotics without a prescription [13]. With α = 0.05, Z_1-α_ = 1.96 and a precision of d = 5%, we calculated that at least 246 drug transactions needed to be observed. In a pilot study, we observed that there were approximately five to six antibiotic transactions per pharmacy per day in a rural pharmacy. Hence, we estimated that we needed to observe at least 15 drug stores in 3 consecutive days in the rural area. We selected an equal number of urban and rural pharmacies to facilitate comparison although urban sales are expected to be considerably higher.

### In-pharmacy observation

During three consecutive days from opening time to closing time of the pharmacy, investigators observed and recorded all information related to pharmacy and drug selling practices onto data capture forms. The forms captured the following basic pharmacy data: facilities, number of staff and education level, presence of Good Pharmacy Practice (GPP) certificates, and presence of pharmaceutical guidelines. Pharmacies that have a GPP certificate are required to ensure a supply of high quality healthcare products and deliver sufficient information and advice to the consumer. The GPP policy also requires pharmacies to have proper facilities (area, drug storage), and comply with prescription regulation.

For the observation of drug transactions, we captured the following information: gender, estimated age of customer, indication for buying drugs (coded according to the International Classification Primary Care – ICPC edition 2), presence of a prescription, compliance to prescription, and any advice provided by drug seller. In cases in which a prescription was provided we checked whether the drug on the prescription was substituted by another drug with a different generic name or a different content/concentration than on the prescription was dispensed or a different dosage/duration than on the prescription was dispensed. In case any of the above was done, we then determined that there was non-compliance with the prescription.

A “drug transaction” in this study included the purchase of any drug or other items present in the pharmacy (e.g. herbal medicine, cotton wools, band aid, etc.). Purchased drugs were recorded according to brand name which was subsequently recoded into the corresponding generic name and Anatomical Therapeutic Chemical (ATC) Classification System. For each drug we also recorded the origin, unit, dosage, and selling price.

The observers included pharmacists who recently graduated from Hanoi Pharmacy University, master pharmacy students, and trained field workers. They were trained in observation skills, interview skills and how to complete the capture forms by senior and experienced investigators. The training included presentation and explanation of the study, discussion, interaction and case practice by acting as drug sellers/owners and interviewers. Furthermore we performed a pilot in two pharmacies (one in urban, one in rural) to test the questionnaire and revise if needed. The pilot pharmacies were not selected for the real observation.

The pharmacist/seller was informed that the observation would be for all drug sales, and thus not antibiotics specifically, to reduce any potential biases by the observation. The observations were supervised and randomly checked by supervisors. At the end of each observing day, supervisors collected the forms and were checked for completeness. Bigger pharmacies had two observers present. Pharmacy customers were not interviewed by the study staff.

All data capture forms and questionnaires were designed in the English language and send for peer review to experts in the field. The revised version was then translated into Vietnamese and piloted in a rural and urban pharmacy.

### Post-observation questionnaire and qualitative assessment

After the observation, one drug-seller and one pharmacy owner per pharmacy were asked to complete a semi-structured questionnaire which focused on antibiotic sales and their opinions about important causes for irrational antibiotics dispensing in their region. Answers were provided on a 5-point likert scale: “1 = strongly disagree” to “5 = strongly agree”. To assess the reliability of survey responses, Cronbach’s alpha was analyzed with respondents’ scores for all questionnaire items by SPSS. It is a coefficient from 0–1, with values above 0.7 being acceptably consistent.

All forms were anonymous to encourage interviewees to frankly share information. In total, 43 informants attended this survey including 26 respondents in urban pharmacies and 17 in rural site. Among them, 4 respondents in urban and 13 in rural were both pharmacy owners and sellers.

Qualitative methods, focus group discussions (FGD) and in-depth interviews, were used to better explore experiences and opinions of the drug sellers and pharmacists, as well as their perceptions of the factors that impact on inappropriate antibiotic dispensing. One FGD was held in the rural area and a total of six individual in-depth interviews were performed in both sites due to difficulties in finding participants for the FGD, especially in urban area. The FGD included the following participants: two pharmacy owners, one drug seller and three commune health center workers. All of them had a primary degree on pharmacy and those working in commune health center (CHC) were also assistant doctors. The in-depth interviews were done with three rural (two pharmacy owners and one CHC staff) and three urban participants (two participants were both a pharmacy owner and drug sellers and one drug sellers, one owner is pharmacist and two other participants had a secondary degree on pharmacy). English guidelines were developed to cover general and specific issues for asking participants to discuss their own experiences and opinions.

Both discussion and in-depth interviews included the following themes: (1) financial incentives, (2) knowledge of government regulations and (3) potential solutions for controlling inappropriate antibiotic dispensing practices (see Additional file 1: Table S1). All discussions in both sites were led by NQH who had relevant training and experience. If needed, findings of the observation and questionnaire were presented during FGD and interviews to support the discussion. All contents of conversations were recorded and transcripts were made and translated into English. Data from transcripts were analyzed using qualitative content analysis by listening to the tapes and reading and re-reading the transcripts to become familiar with the data and to categorize information. We used both the Vietnamese transcript and the English translated version to identify common themes. Connections within and between themes were identified. The main themes and connections were used to identify important causes of inappropriate antibiotic dispensing in urban and rural pharmacies [17].

### Ethical considerations

The Ethical Review Board of Hanoi Medical University approved the study (Decision No: 78/HDDD-YHN). Permission was obtained from the local health bureau for the study and verbal consent was obtained from the owner of each participating pharmacy. All pharmacy data was anonymized.

### Data analysis

Collected data were cleaned and entered into a database and checked for quality by an independent data analyst. Antibiotic sales data was summarized using median and interquartile range (IQR) for skewed distributed data. Potential differences between urban versus rural pharmacies were compared by Mann–Whitney test for non-normal continuous data, Wilcoxon sign rank test for paired non-normal data and Chi-square test for categorical variables. P-values less than 0.05 were considered significant (2 tailed).

In term of revenue, the contribution of antibiotic sales to the total drug sales for each pharmacy was calculated. In addition, we calculated the retail mark-ups of the twenty most sold antibiotics. The mark up is the difference between the cost and selling price of a particular product. Here, we used the percentage mark up to assess the profit of antibiotics that was calculated as (selling price – purchasing price)/purchasing price × 100%. The purchase prices were obtained from major wholesalers and distributers in northern Vietnam. Data was analyzed by SPSS software, version 16 (SPSS Inc., USA). The currency exchange rate of Vietnam Dong (VND) to US dollar (USD) at the time of study was: 1 USD = 18,500 VND.

## Results

### Pharmacy characteristics

Among thirty randomly selected pharmacies, six urban pharmacies had a Good Pharmacy Practice (GPP) certificate, whilst none of the rural pharmacies had a GPP certificate.

None of the private pharmacy owners in the rural area were pharmacists, whereas 5 owners of urban pharmacies were pharmacists. Most urban pharmacies had two drug sellers working in each store while rural pharmacies usually had one seller per outlet. Drug sellers in the urban pharmacies had a higher level of education: 3/28 had a university degree on pharmacy, 21/28 were assistant pharmacists, and 4/28 had an elementary degree on pharmacy. In the rural pharmacies none were pharmacist, 9/17 were assistant pharmacists, 4 were elementary pharmacists and 4 were doctor assistants. Three urban pharmacies had a pharmacist on site in charge of managing and dispensing drugs. Only one pharmacy had up-to-date reference books available in the pharmacy and frequently used for consultation, the remaining did not (see Additional file 2: Table S2).

### Observation of drug sales

In total 2953 drug sale transactions (2083 urban and 870 rural) were observed between the 30 pharmacies (Table 1). The proportion of transactions that included antibiotics was high: 24% (499/2083) in the urban sites and 30% (257/870) in the rural sites (p = 0.002). Most antibiotics were sold without a prescription: 88% (439/499) in urban and 91% (234/257) in rural area (p = 0.2 showing no significant difference between two areas). Compliance to regulations was better in the pharmacies that had a pharmacist on site with 19% (21/112) of total antibiotics transactions having prescription versus only 10% (62/644) in the shops without pharmacist (p = 0.004).

**Table 1:** Antibiotics dispensing practices according to prescription regulation

**Outcomes**	**Urban (n = 2083)**	**Rural (n = 870)**
Transaction with antibiotics	499 (24%)*	257 (30%)*
*With prescription*	60 (12%)	23 (9%)
Comply with prescription	49 (82%)	18 (78%)
Not comply with prescription	11 (18%)	5 (22%)
*Without prescription*	439 (88%)	234 (91%)
Client made decision	221 (50%)*	66 (28%)*
Drug seller made decision	218 (50%)	168 (72%)

*Significant different between urban and rural group using chi-square test (p < 0.05).

There was no significant difference between GPP versus non-GPP pharmacies regarding antibiotics dispensing practices. Pharmacies with a GPP certificate sold antibiotics without prescription in 88% (196/224) of cases, similar to 90% (477/532) (p = 0.38) rate in pharmacies without such a certificate. In term of self-medication, 50% (221/439) of the urban pharmacy customers decided by themselves which antibiotics to buy, whereas the rural clients more often asked for advice from drug sellers, with only 28% self-prescribed (p < 0.0001).

**Figure 1:**
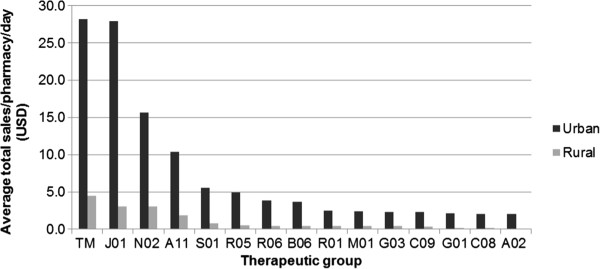
**Average sales in USD per pharmacy per day by therapeutic groups in urban versus rural (in USD).** TM: Herbal medicines, J01: Antibiotics, N02: Analgesic, A11: Vitamins, S01: Ophthalmological, R05: Cough and cold preparation, B06: Hematological agent, R06: Antihistamine, R01: Nasal preparations, M01: Anti-inflammatory and antirheumatic products, G03: genial system, C09: rennin-angiotensin, G01: Gynecological, C08: calcium channel blocker, A02: acid related disorders.

It was observed that antibiotics were the most common drug sold at the pharmacies in both areas (17% in urban and 18% in rural, p = 0.15), followed by herbal medicines (15% in urban and 11% in rural, p < 0.0001). However, in term of monetary value, herbal medicines was the most important groups which mainly contributed to total sales of both urban and rural pharmacies with 24% in urban and 27% in rural, followed by antibiotics (24% in urban versus 18% in rural), analgesics group and vitamins (Figure 1). Average number of customers per pharmacy per day was 46 in urban and 19 in rural area. Among them, 11 clients in urban area had transactions that included antibiotics and the corresponding figure in rural area was 6 clients (Additional file 3: Figure S1). Other therapeutic groups, such as cardiovascular system, nervous system, or corticosteroid medications, were rarely dispensed in all observed pharmacies. Three most common sold antibiotics in the urban area were: amoxicillin (13%), azithromycin (12%), cephalexin (9%) while in rural pharmacies were amoxicillin (27%, p < 0.0001), cephalexin (20%, p < 0.0001) and ampicillin (12% versus 4% in urban setting, p < 0.0001). The main difference between the urban and rural pharmacies was that older antibiotics, such as chloramphenicol, and cotrimoxazole, were more commonly dispensed in the rural area. In the urban area more new and expensive brands such as augmentin (amoxicillin-clavulanic acid), 3^rd^ generation cephalosporins (cefuroxime, cefixime), and azithromycin were sold.

The most common reason for buying antibiotics in the urban sites was cough (32%), and in the rural sites this was fever (22%). Antibiotics were often sold in combination with other drugs: analgesics 17% (189/1122), cough and cold preparations 16% (182/1122), vitamins 9% (99/1122), corticosteroids 9% (98/1122), and herbal medicines 5% (54/1122).

### Economic indicators of antibiotic sales

Antibiotics represented a considerable proportion of total revenues per day: 24% (27.9USD/115.8USD) in urban and 18% (3 USD/16.5 USD) in rural area (p = 0.59) (Figure 1). Urban pharmacies showed higher sales of imported antibiotics with median sale of 11.5 US dollars per pharmacy per day (IQR = 5.3 – 41.7) compared to domestic antibiotics (median = 5.1 US dollars, IQR = 4.2-6.6, P-value 0.003). The opposite was observed in the rural area where very little imported products were sold with median sales of zero US dollars per pharmacy per day compared to domestic products in term of total antibiotics monetary sales with median sale of 1.6 US dollars (IQR = 1.4-3.1), p value < 0.001). In the rural sites, available imported brands such as amoxicillin or cephalexin were mostly from India, with relatively low prices as compared to other brands. Meanwhile, more expensive imported brands were preferred by urban customers.

Retail mark-ups of twenty most common sold antibiotic generics across all pharmacies in each setting varied considerably. In the urban area, mark-ups ranged from 17-243% (median = 54%, IQR = 30-79%) and in the rural area from 21-186% (median = 58.5%, IQR = 39-67%). There was no significant difference between the mark ups between the two regions (p = 0.76). Several imported brands that were only dispensed in urban pharmacies showed relatively high mark-ups such as: augmentin (amoxicillin – clavulanic acid), zinnat (cefuroxime), zithromax (azithromycin) as compared to domestic products (Table 2).

**Table 2:** Mark-ups of 20 most common sold antibiotics according to generic names

**Generic name**	**Origin**	**ATC code**	**Unit/Content**	**Unit price (USD) USD**	**% Mark-up**	
					**Urban**	**Rural**
Amoxicillin	Vietnam	J01CA04	Tablet/500 mg	0.02	78	67
Amoxicillin	India	J01CA04	Tablet/500 mg	0.04	54	54
Amoxicillin	Austria	J01CA04	Tablet/500 mg	0.04	88	NA
Cephalexin	Vietnam	J01DA01	Tablet/500 mg	0.04	67	67
Cephalexin	India	J01DA01	Tablet/500 mg	0.05	58	58
Cephalexin	France	J01DA01	Tablet/500 mg	0.07	25	NA
Ampicillin	Vietnam	J01CA01	Tablet/500 mg	0.02	122	43
Ampicillin	India	J01CA01	Tablet/500 mg	0.03	100	40
Chloramphenicol	Vietnam	J01BA01	Tablet/250 mg	0.03	17	59
Cotrimoxazole	Vietnam	J01EC01	Tablet/480 mg	0.01	33	67
Metronidazole	Vietnam	J01XD01	Tablet/250 mg	0.01	82	150
Lincomycin	Vietnam	J01FF02	Tablet/500 mg	0.03	41	25
Penicillin	Vietnam	J01RA01	Tablet/1 MIU	0.02	60	50
Spiramycin	Vietnam	J01FA02	Tablet/0.75 MIU	0.04	43	29
Spiramycin	France	J01FA02	Tablet/0.75 MIU	0.14	68	21
Ciprofloxacin	Vietnam	J01MA02	Tablet/500 mg	0.02	233	186
Ciprofloxacin	German	J01MA02	Tablet/200 mg	0.65	33	NA
Erythromycin	Vietnam	J01FA01	Pack/250 mg	0.08	103	69
Erythromycin	France	J01FA01	Pack/250 mg	0.23	31	67
Azithromycin	German	J01FA10	Bottle/200 mg/5 ml	5.19	19	NA
Cefuroxime	UK	J01DA06	Tablet/500 mg	1.06	27	NA
Cefixime	Bangladesh	J01DA23	Pack/200 mg	0.20	49	NA
Amoxicillin+						
Acid clavulanic	UK	J01CR02	Pack/250 mg	0.37	62	NA
Roxythromycin	India	J01FA06	Tablet/150 mg	0.06	81	NA
Klarithromycin	USA	J01FA09	Tablet/250 mg	0.43	31	NA
Tobramycin	Belgium	J01GB01	Vial/0.3%	2.05	18	NA
Cefpodoxim	India	J01DA33	Tablet/100 mg	0.04	19	NA
Clindamycin	India	J01FF01	Tablet/300 mg	0.05	117	NA

NA: Not available.

The semi structured questionnaire on antibiotic dispensing practices with drug sellers and drug store owners by semi-structured questionnaire, 41% (7/17) of rural respondents and 27% (7/26) of urban informants conceded that 20% to 40% of their total profit was due to antibiotic sales (p = 0.33). Meanwhile 53% (9/17) in rural and 23% (6/26) in urban site thought that profit from antibiotics accounted for less than 20% (p = 0.04). Only 6% (1/17) of rural respondents and 4% (1/26) in urban considered that profits from antibiotics accounted for 40-60% of their total profit.

### Causes for inappropriate antibiotic selling

All rural pharmacy respondents thought that the fear of losing a customer leads to dispensing of antibiotics without prescription. This opinion was shared with 69% (18/26) of urban respondents. Pressure from patients that demand antibiotics was considered a significant driver of irrational dispensing practices in rural pharmacies according to 77% (13/17) of respondents, and 39% or urban respondents (p = 0.01). Only 27% (7/26) of the respondents in urban and 24% (4/17, p = 0.8) in rural area considered that knowledge of drug sellers was insufficient to dispense antibiotics appropriately. The majority of urban respondents (69%) thought that inappropriate prescription of doctors contributed to irrational antibiotic selling, whereas trust in doctors appeared stronger among respondents in rural setting (29%, p = 0.01). 31% in urban and 35% in rural sites conceded that inappropriate dispensing of antibiotics to be due to high profitability of antibiotic sales (p = 0.75). 71% (12/17) rural participants blamed inappropriate dispensing on other causes like quality of diagnostics and access to medical services versus 46% (12/26) in urban site (p = 0.12) (Table 3). Only a minority 8% of urban and 18% of rural respondents thought that the current situation of antibiotics dispensing was appropriate and does not need to be improved (p = 0.32).

**Table 3:** Causes for irrational antibiotics dispensing

	**Percentage of respondents within area agreed with given reasons**	
**Reasons outcomes**	**Urban (n = 26)**	**Rural (n = 17)**
Fear of losing customers	18 (69%)	17 (100%)
Pressure from patient’s demand	10 (38%)*	13 (76%)^*^
Insufficient knowledge of dispensers	7 (27%)	4 (23%)
Inappropriate prescribing of doctors	18 (69%)^*^	5 (29%)^*^
High profitability of antibiotics	8 (31%)	6 (35%)
Other (quality of diagnosis or health services)	12 (71%)	12 (46%)

*Significant different between groups using Chi-square test (p value <0.05).

### Qualitative study

#### Incentives structure

Most interviewees in both the urban and rural setting did not think that profits from antibiotic sales predominated in comparison with other drugs. According to their opinion, vitamins, tonic drugs or functional foods are more profitable than the antibiotic group, which, however, is not confirmed by our quantitative data. “Antibiotics are commonly used items and customers know well their prices. That is why it not as profitable as less popular drugs like vitamins, tonics or functional foods” was the response of one rural seller. Nevertheless, they conceded that pharmacy’s income would be affected if they comply with prescription regulation. “Not only antibiotics but also thirty other groups have to be dispensed with a prescription. If we wait for a prescription, we sell hardly anything and total sales would be definitely influenced” according to one urban seller.

All rural interviewees stated that patients’ demand is a common factor affecting the sale of antibiotics. An example of this is described as: “I need to satisfy clients’ demand. That’s in the interest of our business!”. According to their opinion, this factor can be changed if patients’ awareness is improved and when the knowledge of sellers is strong enough to give professional advice. Meanwhile, fear of losing customers is common among urban sellers. “If I refuse to sell antibiotics without prescription, I will lose that customer for another pharmacy as they can easily buy anywhere”.

Both urban and rural respondents reported that patients often avoided visiting doctors due to the inconvenience, and would rather go directly to a private pharmacy as the first choice for mild disease. “It’s very annoying and time-consuming to be examined in a hospital. And private clinic are very costly, as they do many kinds of test. Our customers only go to see doctors in case of severe disease”.

### Knowledge on antibiotics/resistance and regulations

All urban and rural participants expressed that they will give antibiotics in case of suspected infection such as upper respiratory infections with fever, cough and sputum or even an injury to prevent infection. In addition, some rural interviewees noted that customers consider antibiotics to be a ‘miracle drug’ that can treat many kinds of diseases and sometimes they demand it simply for maintaining a private stock for self-medication. Meanwhile, all urban interviewees believed that misconceptions about antibiotic use changed among the urban population where there are better economic conditions and higher educational levels. “Recently, public awareness of drugs’ side effects has been improved, so there is less abuse of antibiotics than before” according to an urban seller.

All interviewees stated that they had heard about antibiotic resistance. However, qualitative data also revealed insufficient knowledge of antibiotic resistance among drug sellers and pharmacy owners, especially in the rural area. Most urban drug sellers demonstrated reasonable knowledge regarding the possible effects of resistance on all populations, whereas some rural sellers did not. “Antibiotic resistance occurs in those overusing it. I do not abuse, so for me there is no need to worry” (Rural seller).

Most respondents believed that patient-related factors such as self-medication and poor adherence to antibiotic regimens contribute to the problems of antibiotic resistance. It has been reported in the rural setting that patients often buy antibiotics for an inappropriate duration. “I advised the customer to take antibiotics for at least 5 days, but they do not have enough money so they usually buy for just 2.5 days (10 tablets). When they recover, they will stop taking drugs, otherwise they would have bought more” (Rural seller).

It was also reported that there is not enough attention to antibiotics and resistance in the curriculum of pharmacists and drug sellers.

Regarding the knowledge of government regulations, most rural respondents did not know about GPP. “This is the first time I heard about GPP” said a rural seller. They also revealed misconception about prescription regulation by stating that: “Some kind of weak antibiotics such as amoxicillin or ampicillin can be sold without prescription” (Rural seller). In contrast, all urban interviewee understood clearly about GPP, but they conceded that there is little enforcement in dispensing practice. “There is no difference between GPP and non-GPP pharmacy in terms of regulation compliance. Over the counter dispensing of prescription only drugs is common in every pharmacy” (Urban seller).

### Proposed solutions

Rural respondents did not think that GPP could be deployed broadly in the rural setting due to the poor conditions of the facilities and education level of the work force. However, if regulations are enforced they will shift their business to dispense over the counter drugs like vitamins, cough and cold preparation; tonics that are allowed by the law to compensate pharmacies for financial losses. The urban respondents believe that GPP brings improvement to infrastructure but not to dispensing practices. “To get a GPP certificate, we need to invest more in improving our infrastructure; as a result the pharmacy looks more spacious. However, quality of service and dispensing practices has not been much improved”.

Pharmacy workers have the understanding that the GPP policy objective is to improve the quality of pharmacy services in terms of infrastructure and quality drug supply. However, the awareness about their own professional contribution in promoting rational medicine use and its role in public health is very limited.

Both urban and rural respondents considered that training for drug sellers and the general population was needed to improve their knowledge and awareness about antibiotics and resistance and thought that this would likely have a significant impact on controlling inappropriate antibiotic use in the community. “There will be less pressure to give customers antibiotics if their awareness is improved”.

## Discussion

The results of this study clearly illustrate the widespread inappropriate antibiotic dispensing at private pharmacies in the Hanoi region. With only about 10% complying with prescription regulations, the situation in Vietnam is worse than has been reported in Zimbabwe, where the proportion is 39% [18]. In a cross-sectional client simulation study in Syria, 87% of the pharmacies sold antibiotics without a prescription. This proportion increased up to 97% when the client simulators insisted on buying antibiotics [19]. Similar studies in Saudi Arabia and India had comparable results: 78% and 94% of visited pharmacies dispensed over the counter antibiotics [20,21]. The most frequent reason for buying antibiotics was acute upper respiratory tract infections, which are generally self-limiting [22,23].

There are several successful interventions in other countries that brought important reduction in antibiotic use. As reported in Chile, consumption of most oral antibiotic groups in the community pharmacies significantly decreased after fulfilling prescription-only regulations [24]. Similarly, inappropriate antibiotic prescribing in viral illness remarkable declined as in Korea by prohibiting prescribers from dispensing medications themselves [25]. In Vietnam, prescription-only regulation is embedded in the Drug Law that was issued in 2005 [26]. In spite of these regulations, there is no sanction for non-compliance. This may explain why, to this moment, no pharmacy has been penalized for antibiotic dispensing without a prescription. As there is a lack of enforcement of the regulations, self-medication is possible and is viewed as more economical and convenient than consulting a health professional [27,28]. Even if a pharmacy has a Good Pharmacy Practice registration, the results of this study revealed that the awareness of the concept of GPP among drug sellers was poor and they dispensed antibiotics without a prescription similar to pharmacies without a GPP standard. We also observed that more than 80% of the pharmacies rented pharmacist’s licenses. According to Vietnamese regulations (Decree 79/2006/NĐ-CP), only pharmacists with at least 5 year experience can own a pharmacy [29]. However, pharmacists often rent out their license and work elsewhere, making it easier for non-pharmacists to own a pharmacy. Despite the limited number of pharmacies in our study, we did observe better practices in sites that had a pharmacist present. As health promoter in the situation of being the “front-line health worker”, pharmacist should promote non-drug solutions for any health problems. Strengthening this role of pharmacist in distributing channel might have impact on reducing irrational antibiotics in community.

Antibiotics represented a considerable proportion of total revenues (24% in urban and 18% in rural pharmacies), illustrating that antibiotics sales contribute an important part of total sales of pharmacies. Imported brands were sold more in urban pharmacies, whereas rural pharmacies generally mostly sold domestically produced antibiotics. The study also found that in the urban area, patients’ demands are a common factor affecting the sales of antibiotics, with half (50%) of urban clients self-prescribing. In contrast, clients in the rural sites more often asked for advice from drug sellers. However, lack of knowledge of drug dispensers is common and will not lead to better antibiotic dispensing practices. The qualitative study also disclosed that the government push to have all pharmacies comply with GPP standards is likely not a solution due to lack of enforcement and the shortage of a well-educated workforce [26]. According to the Vietnamese General Statistics Office, in 2010, there were only 0.4 pharmacists/10,000 inhabitants, and for assistant and elementary pharmacists this was about 2 and 0.6 per 10,000 inhabitants. Pharmacy staffs with a university degree mostly work in the big cities with 4.5 pharmacists/1000 inhabitants, despite a serious deficiency in remote areas with only 0.2 pharmacists/10,000 inhabitants [26].

Overuse of antibiotics in the community is caused by people buying antibiotics after self-diagnosis or diagnosis by, often poorly trained, health-care providers. The reasons for irrational antibiotic prescribing in Vietnam are the same as in other countries including perceived expectations of patients, time constraints, lack of knowledge, lack of diagnosis capability and financial benefits for the prescriber [26]. Identifying and modifying the incentives for inappropriate prescribing remains a major challenge.

In term of impact of implementing pricing policies, high prices of antibiotics and tendency to sell branded drugs rather than cheaper generics is one of the important factors affecting irrational use and inadequate treatment as people often cannot afford to buy a full treatment course. The current mechanism of drug price control is not able to achieve the desired objectives as the drug prices in Vietnam are higher as compared to international comparators [30]. The government has no leverage to negotiate on the wholesale prices even if those prices are higher than CIF prices (cost, insurance and freight). Retail prices are determined by the market, but there is a tendency to sell branded drugs rather than cheaper generics in urban areas. According to WHO’s studies in private sector, there was a big variation in mark-ups along the Vietnam medicines supply chain [30]. Suppliers can easily increase prices and the government cannot control this. It is important to have a more structured and enforced price control mechanism, with strong generic policies, good procurement systems and single system leverage (such as health insurance and bulk procurements) to reduce drug prices.

Lastly, it was clearly revealed in both the quantitative and qualitative study that there is poor awareness of consumers. As shared experiences from several developed countries in Europe [31], education campaigns targeting on providers and consumers through mass media contributed to reduction of antibiotic overuse suggesting that public education campaigns can be effective.

There are some limitations to our study that needs to be discussed. Our study was conducted only in the Hanoi region, with a relative small sample size and can therefore not be generalized to the whole country. However, discussions with doctors and pharmacists from other regions, do confirm that the issues are similar elsewhere. In larger pharmacies, some transactions may have been missed when large numbers of customers come to shops simultaneously. However, we believe this was limited as in larger pharmacies, as two observers were present. Awareness of being observed might have influenced antibiotic dispensing practices (Hawthorne effect). To minimize this bias the sellers were unaware during the observation that the objective was to assess antibiotic sales. Questionnaires focusing on antibiotics were done after the observation. Some respondents were both drug seller and pharmacy shop owner, which might affect the results as the owner may mostly be interested in profit and their opinion about incentives driving irrational antibiotic dispensing might be different from a drug seller. There was limited participation for participants in the urban area to join focus group discussions which may account for the relative paucity of solutions. Only one focus group discussion could be performed in the rural setting and in the urban area we conducted in-depth interviews. With relatively few participants in the interview, we were not able to estimate where the saturation was reached. However, at the end of the discussion and interviews, little new ideas were recorded, so we do think we were close to saturation with our limited number of interviews. Furthermore, the study used wholesale prices to assess mark-ups of sold antibiotics as we were unable to obtain purchasing prices from the pharmacies. Finally, the limited observation time of three days in each pharmacy will not reflect the sales of antibiotics and dispensing practices fully as these may be subject to change due to diseases with seasonality (e.g. influenza season). However, we do think the observations nicely reveal the magnitude of inappropriate antibiotic dispensing.

## Conclusion

The revenues from antibiotic sales are considerable for private pharmacies in both rural and urban northern Vietnam. Complying with drug regulations, to dispense antibiotics only with a prescription, would therefore lead to economic loss for pharmacies. This would make acceptance and compliance with regulations challenging. Increasing the requirement for pharmacies to be GPP certified may only help in case the regulation that a pharmacist should be on site is enforced. For non-GPP pharmacies, substituting antibiotic sales with sales of symptom relieving drugs or vitamins may be a strategy to compensate pharmacies for financial losses and to motivate them to comply with government regulations. Confronted with the situation of not enforcing regulations, continuing professional training for drug sellers will be helpful to increase their understanding of antibiotics, resistance and how to dispense it appropriately. As the consumers often demand antibiotics without a prescription, public awareness campaigns should also be a central component of future control strategies.

## Competing interests

We have no potential conflicts of interest to declare.

## Authors’ contribution

We are co-authors in this manuscript and have contributed sufficiently in the work as follow. DTTN: the first author who was responsible for and monitored the observation and in-depth interviews, performed statistical data analysis and drafted the manuscript. NTKC: The second author, who contributed in the planning of the study, supervised the observation period and took part in revising the manuscript. NPH: The third author, who was involved in supervising observation and revising manuscript. NQH: The fourth author, who performed in-depth interviews and revised manuscript. NTTN and HTL: The fifth and sixth authors, who have participated in data collection and statistical analysis. TKT and HDP: The seventh and eighth authors, who have contributed in statistical analysis and manuscript revision. PH: The ninth author, who was participated in designing of the study, NVY, NVK: The tenth and eleventh authors, who have took part in revising manuscript. HFLW: The last author, who was responsible for conception and designing study and manuscript revision. All authors have read and approved the final manuscript.

## Pre-publication history

The pre-publication history for this paper can be accessed here:

http://www.biomedcentral.com/2050-6511/15/6/prepub

## Supplementary Material

Additional file 1: Table S1 Thermalized guideline for FGD and in-depth interview. Click here for file

Additional file 2: Table S2 Pharmacy baseline information. Click here for file

Additional file 3: Figure S1 Average number of clients per pharmacy per day. Click here for file
