# Supplementary material for: Antibiotic sales in rural and urban pharmacies in northern Vietnam: an observational study
Source: BMC Pharmacol Toxicol. 2014 Feb 20;15:6. doi: 10.1186/2050-6511-15-6 (PMC3946644; doi:10.1186/2050-6511-15-6)
Supplement: Additional file 1: Table S1 — Thermalized guideline for FGD and in-depth interview. [file 2050-6511-15-6-S1.pdf]

**Supplementary Table 1. Thermalized guideline for FGD and in-depth interview**

**1) Financial incentives**

a) What is the importance of antibiotic sales in comparison with other drugs for profit in your pharmacy?
b) What factors affect the sales of antibiotics? Can we change these factors or not?
c) Why are antibiotics still sold when there is no prescription?
d) What is role of distributors /prescribers /patients?
e) What type of patients demand antibiotic without prescription and is this common?
f) If a customer requests antibiotics when not needed, would you sell or not?

**2) Knowledge of government regulation**

a) What do you know about antibiotic resistance and the role of inappropriate antibiotic use? How did you acquire this knowledge?
b) Are current government regulations sufficient to control inappropriate antibiotic use?
c) Will Good Pharmacy Practices help to improve control of inappropriate antibiotic use?

**3) Solutions**

a) What would happen if antibiotic sales decline due to compliance with current regulations on dispensing antibiotics?
b) In your opinion, what do we need to do to improve the situation of antibiotic use and resistance in Vietnam?
c) What are the roles and responsibilities of other stakeholders like industry and government?
