# Supplementary material for: Antibiotic sales in rural and urban pharmacies in northern Vietnam: an observational study
Source: BMC Pharmacol Toxicol. 2014 Feb 20;15:6. doi: 10.1186/2050-6511-15-6 (PMC3946644; doi:10.1186/2050-6511-15-6)
Supplement: Additional file 2: Table S2 — Pharmacy baseline information. [file 2050-6511-15-6-S2.pdf]

Supplementary Table 2. Pharmacy baseline information

	Urban	Rural
Pharmacy is GPP licensed	6 (40)	0 (0)
Pharmacy owner's degree		
Pharmacist	5 (33)	0 (0)
Licensed renter	10 (67)	15 (100)
Assistant Pharmacist	9 (60)	10 (67)
Elementary Pharmacist	1 (7)	1 (6)
Assistant Doctor	0 (0)	4 (27)
Number of drug sellers in pharmacy		
1 seller	5 (33)	11 (73)
2 sellers	7 (47)	4 (27)
3 sellers	3 (20)	0 (0)
Drug seller's degree		
Pharmacist	3 (11)	0 (0)
Assistant Pharmacist	21 (75)	9 (54)
Elementary Pharmacist	4 (14)	4 (23)
Assistant Doctor	0	4 (23)
Drug seller uses references / guidelines		
Frequent	1 (7)	0 (0)
Sometimes	4 (27)	6 (40)
Rare	2 (13)	3 (20)
Never	8 (53)	6 (40)

GPP: good pharmacy practice
