# Supplementary figures and images for: Antibiotic sales in rural and urban pharmacies in northern Vietnam: an observational study
Source: BMC Pharmacol Toxicol. 2014 Feb 20;15:6. doi: 10.1186/2050-6511-15-6 (PMC3946644; doi:10.1186/2050-6511-15-6)

**Supplementary Figure 1. Average number of clients per pharmacy per day**

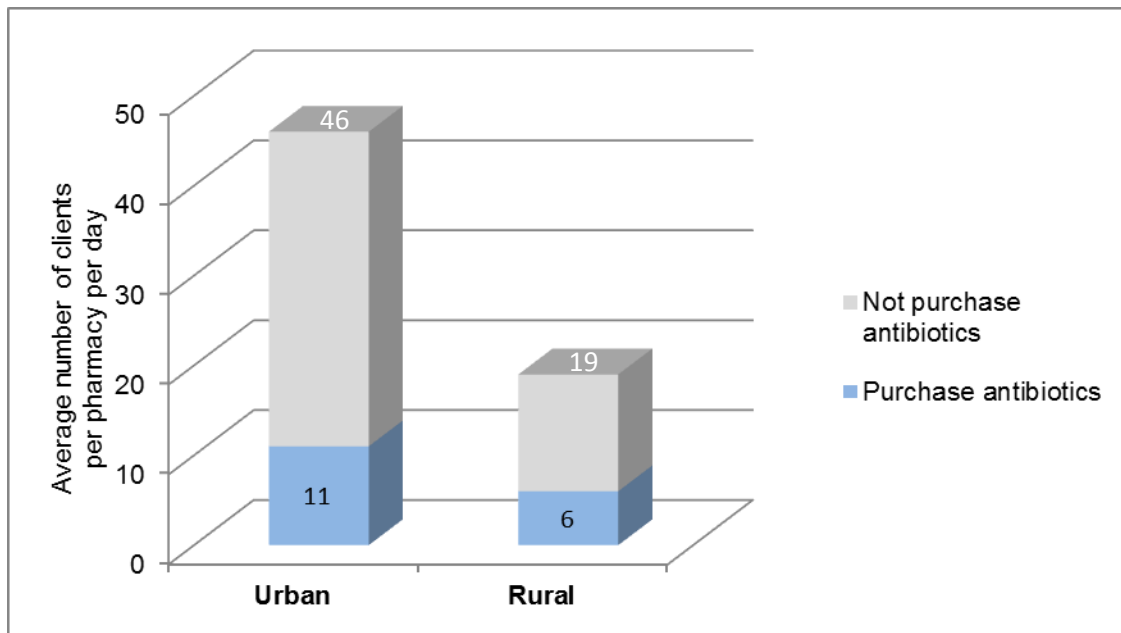

Supplement: Additional file 3: Figure S1 — Average number of clients per pharmacy per day. [file 2050-6511-15-6-S3.pdf]
